# The effects of phytosomal curcumin supplementation on clinical symptoms, and inflammatory and oxidative stress biomarkers in patients with migraine: A protocol for a randomized double-blind placebo-controlled trial

**DOI:** 10.22038/AJP.2022.21242

**Published:** 2023

**Authors:** Mehrnaz Shojaei, Amirhossein Sahebkar, Fariborz Khorvash, Soheil Fallahpour, Gholamreza Askari, Mohammad Bagherniya

**Affiliations:** 1 *Student Research Committee, Department of Community Nutrition, School of Nutrition and Food Science, Isfahan University of Medical Sciences, Isfahan, Iran*; 2 *Applied Biomedical Research Center, Mashhad University of Medical Sciences, Mashhad, Iran*; 3 *Biotechnology Research Center, Pharmaceutical Technology Institute, Mashhad University of Medical Sciences, Mashhad, Iran*; 4 *Department of Biotechnology, School of Pharmacy, Mashhad University of Medical Sciences, Mashhad, Iran*; 5 *Department of Neurology, School of Medicine, Isfahan University of Medical Sciences, Isfahan, Iran*; 6 *Department of Neurosurgery, School of Medicine, Isfahan University of Medical Sciences, Isfahan, Iran*; 7 *Nutrition and Food Security Research Center and Department of Community Nutrition, School of Nutrition and Food Science, Isfahan University of Medical Sciences, Isfahan, Iran *; 8 *Anesthesia and Critical Care Research Center, Isfahan University of Medical Sciences, Isfahan, Iran*

**Keywords:** Curcumin, Phytosomal curcumin, Migraine, Inflammation, Headache

## Abstract

**Objective:**

Migraine is one of the most common diseases. Curcumin with anti-oxidative and anti-neuroinflammatory properties might have beneficial effects in migraine patients. This study will be conducted to evaluate the effects of a phytosomal preparation of curcumin on clinical signs, oxidative stress, and inflammatory parameters in patients with migraine.

**Materials and Methods:**

This is a randomized, double-blind, placebo-controlled, clinical trial in which, 60 patients with migraine will be assigned to receive a daily dose of 250 mg of phytosomal curcumin for 8 weeks (intervention group) or 250 mg maltodextrin as a placebo for the same duration (control group). Before and after the study, frequency, duration, and severity of the attacks, quality of life and sleep, mood status, high-sensitivity C-reactive protein (hs-CRP), Nitric Oxide (NO), and oxidative stress factors will be measured.

**Conclusion:**

It seems that phytosomal formulation of curcumin (a solid dispersion preparation of curcumin with phosphatidylserine) with high bioavailability, can cross the blood-brain barrier (BBB) and result in decreased neuroinflammation, oxidative stress, and neurotoxicity. This way, phytosomal curcumin might lead to reduction of headaches and other complications of migraine and increase the quality of life of patients with migraine.

## Introduction

Migraine as a chronic neurovascular disorder is one of the most common diseases and is considered a primary cause of disability (Agosti, 2018 ▶). It is estimated that the prevalence of migraine is about 12-14% globally and it is more prevalent in women and 35-45 year old individuals (Economics, 2018 ▶; Lipton et al., 2007 ▶). Based on Global Burden of Diseases, Injuries, and Risk Factors (GBD) studies, in 2016, about three billion people suffered from migraine or tension-type headaches. On the other hand, it has been shown that the disability weight of migraine is much higher than tension-type headache, as migraine caused 45.1 million years of life lived with disability (YLDs) while tension-type headache caused only 7.2 million YLDs. Women between ages 15 and 49 years were categorized as the most important age group with migraine headaches (Stovner et al., 2018 ▶). Although it was recently reported that the prevalence of migraine is lower in Asian countries than European countries, North America and Australia (Karimi et al., 2020 ▶), the prevalence of this disease is about 14% in Iran, which is higher than its global range (Farhadi et al., 2016 ▶).

Moderate or severe pain has occurred in about 90% of the migraineurs, and during headache attacks, the ability to function is reduced in about 75% of patients, and others (25%) need bed rest during the attacks (Lipton et al., 2007 ▶). Migraine attacks negatively affect the life quality, and the productivity in both private and social life (Lantéri-Minet et al., 2011 ▶; Farhadi et al., 2016 ▶). It was also revealed that panic and anxiety disorders are dramatically more prevalent among migraineurs compared with others (Sareen et al., 2006 ▶; Jette et al., 2008 ▶). With the increasing frequency of migraine attack episodes, the anxiety rates notably increase. Indeed, findings from a very recent systematic review showed that a major comorbidity of migraine is anxiety, with a mean of ~43% of patients who experienced comorbid symptoms (Karimi et al., 2020 ▶). Another salient fact about migraine is its large economic impact as studies in Europe showed that the average per-person costs for migraine direct and indirect costs were €1222 in a year with a total of €111 billion per year for 27 EU nations (Linde et al., 2012 ▶). In Australia, it is estimated that the cost of migraine was approximately $35.7 billion in 2018 (Economics, 2018 ▶). 

It is proposed that the central nervous system (CNS) is where migraine is originated, while some other evidence suggests that neurovascular and metabolic changes in the brain with dysfunctional intracranial and extracranial blood vessels trigger migraine (Gerring et al., 2018 ▶). Nevertheless, the etiology and pathophysiology of migraine is too complex and not fully understood, and to date, the main cause of migraine attack is unclear (Tajti et al., 2011 ▶). Diverse parameters have been considered migraine triggers such as stress, neuroendocrine imbalances, hormones, oxidative stress, inflammation, too much or too little sleeping, and unhealthy diet and allergenic foods (Hauge et al., 2010 ▶; Finocchi and Sivori, 2012 ▶; Theeler et al., 2010 ▶; Kursun et al., 2021 ▶; Borkum, 2016 ▶). Recent evidence suggests that inflammation through activation of nociceptive sensory neurons has a crucial role in the pathology of pain initiation as well as pain persistence (Sommer and Kress, 2004 ▶). In this regard, an accumulating body of evidence declared that stress, oxidative, and inflammation play a significant role in migraine attacks (Borkum, 2016 ▶; Kursun et al., 2021 ▶; Edvinsson et al., 2019 ▶). The immune system through production of inflammatory factors, namely, cytokines, including tumor necrosis factor (TNF), interleukin (IL) 1 (IL-1), and adiponectin increase headache (Kursun et al., 2021 ▶). Injection of TNF induces headache, while TNF antibody decreases pain in humans (Bruno et al., 2007 ▶). Pro- and anti-inflammatory cytokines increase in the plasma during migraine attacks. Higher level of TNF-α and IL-6 was observed in migraine patients compared with healthy individuals during and between attacks (Yilmaz et al., 2010 ▶). 

Although migraine is becoming one major health issue all around the world, to date, there is no exclusive and comprehensive treatment approach, medical care, or pharmacological agents for its prevention or treatment (Katsarava et al., 2018 ▶). 

Up to the present time, pharmacological care for the treatment of migraine headaches includes triptans, ergot derivatives, and analgesics (NSAIDs). Some other oral drug, originally produced for treatment of epilepsy, depression or high blood pressure, are also applied for the prevention of migraine attacks. Finally, botulinum toxin A injection was approved in 2013 as a preventive therapy in migraine patients who are non-responders to oral preventive medications. Considering several adverse and unfavorable effects as well as the high costs of these medications (D'Amico and Tepper, 2008 ▶; Mayans and Walling, 2018 ▶), and with regard to the facts that patients with severe and/or frequent migraine need long-term preventive medications, several non-pharmacological methods, including nutraceuticals, herbal medicine, behavioral techniques and acupuncture have attracted significant attention to manage migraine in clinical settings (Puledda and Shields, 2018 ▶). Indeed, today, monotherapy remedy is replaced by multiple therapies according to the multiplicity of targets (Mythri and Bharath, 2012). To achieve this purpose, intense investigations are being conducted to assess the combination of modern/conventional pharmacological therapies with medicinal plants, including herbal bioactive compounds and phytochemicals (Kumar, 2006 ▶; Sparreboom et al., 2004 ▶). 

Herbal medicine as a safe, inexpensive, available, and accessible therapy approach is becoming one of the most attractive field of study to prevent and treat neurological disorders such as migraine. Medicinal plants can be considered a complementary and alternative method to prevent and treat migraine since these are an acceptable and favorable therapeutic approach for most of the patients.

Curcumin is a yellow polyphenolic pigment and the principal polyphenol and a key bioactive compound of turmeric, which has been used in traditional medicine for thousands of years. A wide range of beneficial properties are attributed to curcumin (Hewlings and Kalman, 2017 ▶; Bagherniya et al., 2018 ▶; Farhood et al., 2019 ▶; Mortezaee et al., 2019 ▶; Panahi et al., 2014 ▶; Parsamanesh et al., 2018 ▶; Shakeri et al., 2019 ▶; Javandoost et al., 2018 ▶; Kheiripour et al., 2021 ▶; Ghasemi et al., 2022 ▶; Atabaki et al., 2022 ▶). Its neuroprotective properties, as well as its anti-neuroinflammatory effects make it a focus of attraction in the area of neuroscience. Neuroprotective effects of curcumin are attributed to its antioxidant, anti-inflammatory, and anti-protein-aggregate activities (Cole et al., 2003 ▶). Specifically, several animal studies indicated the promising effects of curcumin on neurodegenerative disorders (Park and Kim, 2002 ▶; Zhang et al., 2011 ▶; Frautschy et al., 2001 ▶; Thiyagarajan and Sharma, 2004 ▶). It seems that curcumin might have beneficial effects on migraine signs and symptoms as a very recent systematic review showed that curcumin supplementation had positive effects on the reduction of inflammation and stress oxidative and decreasing the frequency, severity, and duration of migraine attacks (Mohseni et al., 2021 ▶). However, as in this review mentioned, factors that decrease the efficacy of curcumin in the clinical setting are its poor absorption and low bioavailability. It is recommended to use other forms of curcumin such as nano-curcumin, curcumin-piperine, and phytosomal curcumin to overcome the above limitations of the curcumin usage (Mohseni et al., 2021 ▶). 

Curcumin bioavailability increases using the phytosomal formulation of curcumin (a complex of curcumin with phosphatidylserine). Physicochemical properties including amphiphilic nature causes dispersion in both hydrophilic and lipophilic media, which appear with the abundance of phospholipids in phytosomes (Mirzaei et al., 2017 ▶). On the other hand, phosphatidylserine exists in abundant amounts in myelin in the healthy human brain, and its amount in the grey matter increases two times from birth to 80 years of age (Glade and Smith, 2015 ▶). To keep the health of nerve cell membranes and myelin, phosphatidylserine is needed. The absorption efficacy of oral phosphatidylserine is high and it can cross the blood-brain barrier (BBB) subsequent to its absorption into the bloodstream (Glade and Smith, 2015 ▶). 

Altogether, it seems that phytosomal curcumin with high levels of absorption which might cross BBB, could be useful in migraine patients as a novel treatment agent. Different forms of curcumin such as nano-curcumin were previously assessed in migraine patients, and produced promising results in terms of migraine clinical symptoms and also in reduction of inflammation and oxidative stress and the other related factors in these patients (Sedighiyan et al., 2022 ▶; Rezaie et al., 2021 ▶; Abdolahi et al., 2021 ▶; Parohan et al., 2021 ▶). However, to the best of our knowledge, no study evaluated the effects of phytosomal curcumin on migraine patients. Thus, this study will be conducted to evaluate the effects of phytosomal curcumin on clinical outcomes and stress oxidative and inflammatory parameters in migraine patients. 

## Materials and Methods

**Figure 1:**
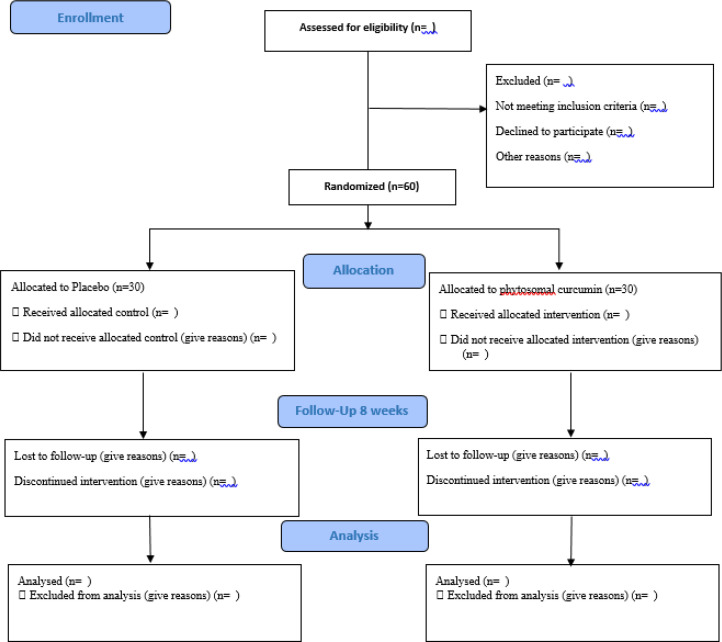
Study Flow Diagram

This protocol was written based on CONSORT SPIRIT 2013 guidelines (Chan et al. 2013 ▶). This study is a parallel double-blind randomized clinical controlled placebo trial in which, a total of 60 migraine patients will be included. The trial design is illustrated in Figure 1.  Patients will be chosen from neurology clinics (Imam Moosa, Sadr, and Khorshid) affiliated to Isfahan University of Medical Sciences from July 2022. To include in the study, subjects will be screened by an experienced neurologist according to our inclusion and exclusion criteria. 

**Eligibility criteria**

Participants will be included in the study according to the following criteria: 

**Inclusion criteria**

a) migraine without aura, migraine will be diagnosed by an expert neurologist (FK) based on ICDH-3 criteria (Munoz-Ceron et al., 2019 ▶) b) history of migraine for at least one year, c) age between 20–60 years old, d) following routine and steady treatment for the management of migraine headaches for at least 4 weeks before of the study, and e) willingness to participate in the study and fulfil the written consent form

**Exclusion criteria**

a) Having other types of headache such as tension-type headache, cluster headache, medication overuse headache, trigeminal autonomic cephalalgias, or headache due to menstrual, b) Pregnancy or lactation, c) history of chronic diseases (i.e. diabetes, high blood pressure, gastrointestinal (GI) disorders like Crohn’s and ulcerative colitis, cancer, or liver, kidney, or thyroid disease), d) any changes in the pharmacological treatment such as type and dose of the drugs, e) Migraine with aura, f) Other neurological disorders, g) taking antioxidant supplements for at least 3 months before the study, h) Following a special diet for at least 3 months before the study, i) Having allergies to herbal medicine, particularly to turmeric and ginger, j) Smoking and alcohol consumption, or k) Individuals with poor compliance to the intervention (less than 80%).

**Randomization**

Eligible patients who fulfill the inclusion criteria will be enrolled in the study. At first, the included patients will be randomized (1:1) into intervention or control groups. We will use a permutated block randomization approach to stratify subjects according to migraine severity with a block size of 4. We will use a table of random numbers to perform random assignment. In addition, patients will be enrolled and assigned into intervention and control groups by a well-trained nutritionist. Allocation concealment will be performed by sequentially numbered containers. 

**Intervention**

Patients in the intervention group will receive one capsule/day containing 250 mg phytosomal curcumin (250 mg containing 20% curcuminoids and 20% phosphatidylserine; Indena SpA, Milan, Italy), for 8 weeks. On the other hand, patients in the control group will receive one capsule/day containing 250 mg maltodextrin, for the same duration. Participants will be asked to take each capsule one hour after breakfast. Moreover, we will instruct the patients in both groups to follow their usual diet and physical activity as well as their medication therapy exactly as their neurologist prescribed. 

**Blinding**

Capsules (curcumin and placebo) will be labeled A and B by the company in the same format of the packages; in addition, capsules will be manufactured in the same shape, size, color and odor. Investigators, patients, laboratory staff, outcome assessors, and data analyzers will be blinded to treatment assignment until final analyses will be conducted. 

**Ethics approval**

The whole protocol has been approved and accepted by the ethics committee of Isfahan University of Medical Sciences (IR.MUI.RESEARCH.REC.1400.110). The study is registered in the Iranian Registry of Clinical Trials, IRCT (IRCT20201129049534N2). All patients will be asked to fill the written consent before being included in the study. We will store data of the participants at the security site. We also will use a unique ID number for each patient for the collected data, laboratory specimens, and reports. Just the research teams will have access to the collected data during the course of the research and these will be remained strictly confidential. The corresponding author will also have access rights to the dataset. In addition, to enable international prospective meta-analyses, the corresponding authors will share the anonymous data with other researchers. 

**Safety consideration**

Curcumin is safe even at high concentrations as it has been previously shown that administration of 8,000 mg curcumin/day for 3 months was safe without any toxicity. Just some gastrointestinal discomfort, specifically nausea and diarrhea was occurred (Hsu and Cheng, 2007 ▶; Chainani-Wu, 2003 ▶). Thus, no considerable adverse effects will be anticipated for the consumption of curcumin and placebo capsules at the mentioned doses. However, if minor side effects occur, it will be reported to the Ethics Committee of Isfahan University of Medical Sciences for decision-making.

**Power calculation and sample size estimates**

Sample size was calculated using the formula suggested for randomized clinical trials considering the type I error of 5% (α = 0.05) and Type II error of 20% (β = 0.2; power = 80%). Nitric oxide (NO) level was also considered a main outcome, and based on a previous study (Zareie et al., 2020 ▶), sample size was calculated 23 persons for each group. With potential dropout, finally 30 patients will be assigned in each group.

**Outcome assessment**

After screening based on the eligibility criteria, participants will be asked to complete a sociodemographic questionnaire including sex, age, education, medical history, drugs, and food supplements (Table 1). To determine the dietary intake of participants to identify energy, and macro- and micronutrient intake relative to migraine, we will use a 3-day food record, assessing two weekdays and one weekend. A well-trained nutritionist blind to the study protocol will instruct participants about how to fill 3-day food records. Food records will be obtained at baseline and after 8 weeks of the intervention. Then, the data will be entered into the Nutritionist IV software to calculate the energy and nutrient intake of the patients. A 24-hour recall will be also used to determine the level of physical activity of the study participants. 

**Migraine assessment**

The migraine headache characteristics including monthly frequency and duration of the attacks will be obtained by a neurologist (FH). Then, patients will be asked to fill questionnaires in terms of frequency and duration of the attacks during the intervention. The severity of pain will be evaluated applying a visual analogue scale (VAS) ranging from 0 to 10. If patients experience no pain, they will instruct to fill 0 and if they suffer from agonizing pain, they will instruct to fill 10 (Zareie et al., 2020 ▶). The Headache Impact Test-6 (HIT-6) scale will also use to assess the degree of disability due to migraine at baseline and at the end of the study. This questionnaire has six questions and examines the effect that migraine has on a person's daily life as a score. In the answer to each question, there are 5 options including never, rarely, sometimes, most often, and always, which have scores of 6, 8, 10, 11, and 13, respectively. The validity and reliability of this questionnaire have been confirmed in a previous study (Ghorbani and Chitsaz, 2011 ▶).

**Assessment of quality of life**

The Migraine-Specific Quality of Life (MSQ) questionnaire will be used to assess the quality of life of migraine patients. The questionnaire consists of 14 questions that assess the quality of life of patients in the last month. The validity of this questionnaire in Iran has been examined by Zandifar et al. (Zandifar et al., 2013 ▶). For each question, a score of one (never) to six (always) is considered, then the sum of the scores of the questions, which is from 14 (minimum total) to 84 (maximum total), will be considered. The sum of the initial scores will be converted to a scale of zero to one hundred, and finally a higher score will indicate a higher quality of life (Zandifar et al., 2013 ▶). To convert the initial score from zero on one hundred scale, we will follow this method: Individual score minus 14 (lowest total score) divided by 70 (distance between the lowest (14) and the highest total score (84)) multiplied by 100. 

**Assessment of mood status (stress, anxiety, and depression)**

A modified DASS-21 questionnaire for Iran will be used to assess mood at the beginning and end of this study (Samani and Joukar, 2007). The DASS-21 subscales consist of 21 questions, seven questions for stress, seven for anxiety, and seven for depression. Each question is scored from zero (does not apply to me at all) to 3 (absolutely applies to me). The subscales should be doubled, then the severity of the symptoms can be determined as shown in Table 2 (Lovibond and Lovibond, 1995 ▶).

**Table 1:** Timeline and applied tests

**Activity**	Time (Week)								
	0	1	2	3	4	5	6	7	8
**Enrollment**	*								
Eligibility screening Informed consent Randomization Allocation	*								
	*								
	*								
	*								
Sociodemographic	*								
Intervention		*	*	*	*	*	*	*	*
Placebo (maltodextrin, 250 mg)		*	*	*	*	*	*	*	*
Intervention (250 mg phytosomal curcumin)		*	*	*	*	*	*	*	*
Compliance and side effects		*	*	*	*	*	*	*	*
Laboratory tests	*								*
VAS	*								*
HIT-6	*								*
MSQ	*								*
DASS-21	*								*
Food record	*								*
Physical activity recall	*								*

**Table 2:** Intensity of each subscale for assessment of mood status

**Severity **	**Depression**	**Anxiety**	**Stress**
**Normal**	0-9	0-7	0-14
**Mild**	10-13	8-9	15-18
**Medium**	14-20	10-14	19-25
**Intense**	21-27	15-19	26-33
**Very intense**	+28	+20	+34

**Assessment of sleep quality**

It was previously shown that episode migraine has an association with migraine (Alstadhaug et al., 2007 ▶; Vgontzas and Pavlović, 2018 ▶), so, we will assess the sleep quality of patients before and after the study using Pittsburg Sleep Quality Index (PSQI) that was previously validated for Iranian population (Farrahi Moghaddam et al., 2012 ▶). The Pittsburgh questionnaire consists of 9 questions that assess bedtime, waking hours, the amount of sleep per day, and other issues related to sleep patterns and mental quality. Each question will be scored between 0 and 3 (0 = not in the past month, 1 shows <1 time in a week, 2 indicates once or twice a week, and 3 shows ≥3 times a week).

**Laboratory assessment**

A well-trained phlebotomist will collect a 10 ml blood sample after 12 hours of fasting from each subject before and after the study. Then, the blood samples will be centrifuged for 10 min at 2500 rpm at room temperature. Enzyme-linked immunosorbent assay (ELISA) will be used to measure the serum levels of serum high-sensitivity C-reactive protein (hs-CRP), Total Antioxidant Capacity (TAC), Total Oxidant Status (TOS), Malondialdehyde (MDA), Superoxide dismutase (SOD), and Nitric Oxide (NO) (KiaZist, Hamedan, Iran). 

**Statistical methods**

The analysis will eventually be performed in the form of Intention-To-Treat (ITT) and Per-Protocol (PP) using the SPSS software, version 22 (SPSS Inc, Chicago, IL, USA). Quantitative data will be reported as mean±standard deviation (SD), and qualitative data will be presented as frequency and percent. Normality of the data will be assessed using Kolmogorov-Smirnov test. Paired t-test will be applied to evaluate the differences in each group before and after intervention. Independent T will be used to show the baseline and endpoint differences between the two groups. We will apply analysis of covariance (ANCOVA) to show differences between two treatment groups after adjusting for confounding variables. P-value less than 0.05 will be considered statistically significant. 

## Discussion

This study is the first clinical trial assessing the effects of phytosomal curcumin on migraine symptoms and complications. Considering the high prevalence of migraine and its several complications, it is indispensable to find novel and safe treatment strategies to reduce pain and migraine attacks, which might result in increased quality of life and improved functional capacity of patients with migraine. Thus, considering the diverse range of beneficial effects of curcumin on different aspects of human health, particularly its promising effects on neurological disorders (Mohseni et al., 2021 ▶), the findings of this trial might be useful in the management of patients in the clinical setting. 

**Figure 2:**
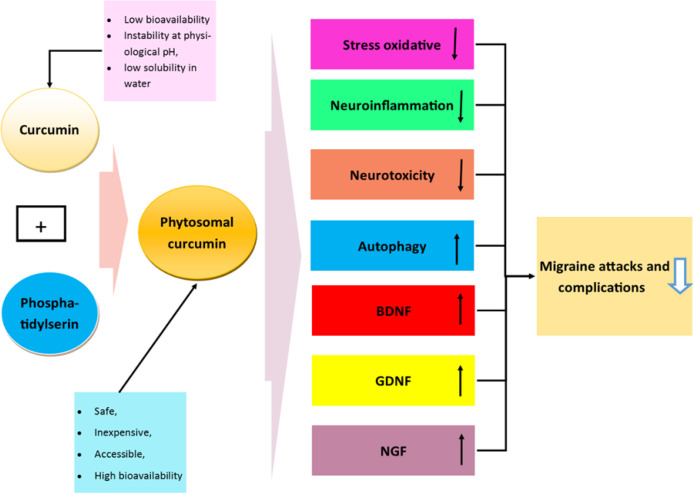
Schematic summary of pathways depicting the possible effects of phytosomal curcumin on migraine and its potential related mechanisms. As shown in the figure, curcumin, as a natural, accessible, safe, and inexpensive phytochemical has some limitations including low bioavailability, instability at physiological pH, and low solubility in water; thus, its combination with phosphatidylserine can effectively reduce the limitations of using curcumin. Phytosomal curcumin might have beneficial effects on migraine attacks and complications through several mechanisms, including reduction in oxidative stress, neuroinflammation, and neurotoxicity. On the other hand, it increases autophagy and brain-derived neurotrophic factor (BDNF), nerve growth factor (NGF), and glial cell line-derived neurotrophic factor (GDNF), which all causes growth, maturation (differentiation), and maintenance of nerve cells (neurons) and promote survival of these cells

In animal migraine models, nitric oxide concentrations were reported to be reduced in response to curcumin administration, suggesting this reduction is mediated through the antioxidant and antinociceptive effects of curcumin (Bulboacă et al., 2017 ▶; Bulboacă et al., 2019 ▶). Inflammation and oxidative stress play significant roles in the pathogenesis of migraine (Parohan et al., 2019 ▶). Importantly, as previously documented, the antimigraine properties of curcumin might be related to its anti-inflammatory and neuroprotective properties (Mohseni et al., 2021 ▶). Indeed, the antioxidant effects of curcumin are mediated directly through reactive oxygen species (ROS) scavenging and indirectly through induction of the expression of antioxidant/detoxifying enzymes and scavengers including catalase, superoxide dismutase, glutathione peroxidase, and HO-1 *via* a Nrf2-dependent pathway (Tapia et al., 2012). Furthermore, anti-inflammatory properties of curcumin are due to its role in the activation of peroxisome proliferator-activated receptor-γ (PPAR-γ) and inhibition of NF-κB signaling pathway (Jacob et al., 2008). Moreover, curcumin decreases neurotoxicity while increases autophagy and brain-derived neurotrophic factor (BDNF), nerve growth factor (NGF), and glial cell line-derived neurotrophic factor (GDNF). These all cause growth, maturation (differentiation), and maintenance of neurons, and promote survival of these cells (Mohseni et al., 2021 ▶; Borkum, 2018 ▶; Allen et al., 2013 ▶), which might lead to the reduction of migraine pain and other related complications (Figure 2).

Beneficial effects of curcumin phytosomes against a variety of conditions including diabetic microangiopathy and retinopathy, cancer, osteoarthritis, and inflammatory diseases have been documented (Mirzaei et al., 2017 ▶), hence, it seems that in our study it might exert promising effects on migraine patients. 

Although this study will be the first randomized double-blind clinical trial investigating the effect of phytosomal curcumin on migraine, some limitations such as relatively short duration of the intervention and lack of long-term follow-up should be acknowledged. In addition, considering the ethical issues, the effect of phytosomal curcumin as a monotherapy approach will not be feasible to be assessed.

The protocol for a double-blinded placebo-controlled trial design is described here, in which the effects of phytosomal curcumin supplementation will be assessed on clinical symptoms as well as inflammatory and oxidative stress biomarkers of patients with migraine. It is expected that oral supplementation with 250 mg/day of phytosomal curcumin for 8 weeks will result in relieving migraine signs and symptoms as well as reduction of inflammation and oxidative stress. Findings of the current study will provide evidence-based information in terms of the efficacy of curcumin as a complementary treatment in patients suffering from migraine.

## Conflicts of interest

The authors have declared that there is no conflict of interest.
